# Evaluation of contaminated drinking water and preterm birth, small for gestational age, and birth weight at Marine Corps Base Camp Lejeune, North Carolina: a cross-sectional study

**DOI:** 10.1186/1476-069X-13-99

**Published:** 2014-11-20

**Authors:** Perri Zeitz Ruckart, Frank J Bove, Morris Maslia

**Affiliations:** Division of Toxicology and Human Health Sciences, Agency for Toxic Substances and Disease Registry, 4770 Buford Highway, MS F-58 Atlanta, GA 30341 USA; Division of Community Health Investigations, Agency for Toxic Substances and Disease Registry, 4770 Buford Highway, MS F-58, Atlanta, GA 30341 USA

**Keywords:** Low birth weight, Preterm birth, Small for gestational age, Volatile organic compounds, Solvents, Military exposures

## Abstract

**Background:**

Births during 1968-1985 at Camp Lejeune were exposed to drinking water contaminated with trichloroethylene (TCE), tetrachloroethylene (PCE), and benzene.

**Methods:**

We conducted a cross-sectional study to evaluate associations between residential prenatal exposure to contaminated drinking water at Camp Lejeune during 1968–1985 and preterm birth, small for gestational age (SGA), term low birth weight (TLBW), and mean birth weight (MBW) deficit. Birth certificates identified mothers residing at Camp Lejeune at delivery. We analyzed exposure data for the entire pregnancy and individual trimesters. For each period examined, births were categorized as unexposed if mothers did not reside at Camp Lejeune or if their residence on base received uncontaminated drinking water. Ground water contaminant fate/transport and distribution system models provided monthly estimated contaminant levels at residences. For PCE and TCE, the exposed group was divided into four levels: < median value, ≥ median value, ≥75^th^ percentile, and ≥90^th^ percentile. For benzene, the exposed group was categorized as <1 part per billion (ppb) versus ≥1 ppb because of sparse data. Magnitude of effect estimates and exposure response relationships were used to assess associations. Confidence intervals (CIs) indicated precision of estimates.

**Results:**

For the highest TCE exposure category during the entire pregnancy, odds ratios (ORs) were 1.5 (95% CI: 1.2, 1.9) and 1.3 (95% CI: 0.8, 2.2) for SGA and TLBW, respectively, and reduced MBW β = −78.3 g (95% CI: −115.0, −41.7). The OR =1.3 (95% CI: 1.0, 1.6) for preterm birth and the highest PCE exposure category during the entire pregnancy. Monotonic exposure-response relationships were observed for benzene exposure during the entire pregnancy and TLBW (highest category OR =1.5, 85% CI: 0.9, 2.3). Although a monotonic association between benzene and adjusted MBW difference was also observed (highest category β = −36.2 g, 95% CI: −72.3, −0.1), the association disappeared when TCE was also added to the model. We found no evidence suggesting any other associations between outcomes and exposures.

**Conclusion:**

Findings suggested associations between in utero exposures to TCE and SGA, TLBW and reduced MBW; benzene and TLBW; and PCE and preterm birth.

**Electronic supplementary material:**

The online version of this article (doi:10.1186/1476-069X-13-99) contains supplementary material, which is available to authorized users.

## Background

The United States Marine Corps (USMC) Base at Camp Lejeune, Onslow County, North Carolina began operations during the early 1940s. Volatile organic compounds (VOCs) were detected in some wells in two of the base’s water distribution systems (Tarawa Terrace [TT] and Hadnot Point [HP]) during the base’s 1980–85 sampling program. Supply wells of a third water distribution system, Holcomb Boulevard (HB), were not contaminated during this sampling period.

The TT system was primarily contaminated with tetrachloroethylene (PCE) from solvent waste disposal from an off-base dry cleaning business [1]. In 1953, the off-base dry cleaning business began disposing of solvent waste in an existing septic tank soil-adsorption system, and it continued disposing of solvent waste in the septic tank through 1985. In 1952, the base began using a supply well for family housing at TT that was approximately 900 feet from the site of the dry cleaning firm [1]. The maximum amount of PCE detected in the distribution system was 215 parts per billion (ppb) in February 1985. Trichloroethylene (TCE), trans-1,2-dichloroethylene (DCE) and vinyl chloride were also present in the TT distribution system due to degradation of PCE in the ground water. The HP system was primarily contaminated with TCE from leaking underground storage tanks, industrial area spills, and waste disposal sites. The maximum amount of TCE detected in the distributions system was 1,400 ppb in May 1982. Vinyl chloride and DCE were present in the HP distribution system due to degradation of TCE. Other major contaminants in the HP system included PCE and benzene [2].

Before delivery to residences, water from contaminated and uncontaminated wells was mixed at water treatment plants. Contamination levels in the drinking water distribution system varied depending on the wells being used at a particular time. The most highly contaminated wells in the HP and TT systems were shut down by February 1985.

The HP system began operation in 1942 and the TT system began operation in 1952. In June 1972, the HB treatment plant began operations and provided drinking water to a service area previously supplied by the HP system. Prior to June 1972, the HB service area was supplied by the HP system. However, during dry weather conditions in the spring/summer months, water from the HP system supplemented the HB system. In addition, the HP system supplied water to the HB system during January 27-February 7, 1985 when the HB system was shut down for repairs. No organic solvent contamination was detected in drinking water from other treatment plants serving the base.

Literature on associations between preterm birth and fetal growth retardation and maternal exposure to solvents in drinking water is limited and inconsistent [3–7]. A study in the Cape Cod region in Massachusetts found no association between prenatal PCE exposure and birth weight or gestational age among children whose mothers were exposed to PCE-contaminated public drinking-water supplies [3]. A study conducted in Woburn, Massachusetts, found that low birth weight and preterm birth were not associated with maternal exposures to public drinking wells contaminated with TCE [5, 6]. However, an OR of 1.6 (95% CI: 1.0, 2.4) was reported for maternal exposure to TCE-contaminated public drinking water during the 3^rd^ trimester and being small for gestation age (SGA). While a study in Tucson, Arizona found no association between TCE-exposed births and full-term low birth weight or low birth weight among all births, an OR of 3.3 (95% CI = 0.5, 20.6) was observed for very low birth weight and TCE [7].

The purpose of this study is to determine if maternal exposures to contaminants in drinking water at Camp Lejeune were associated with preterm birth and fetal growth retardation as measured by reduced mean birth weight (MBW), term low birth weight (TLBW), and SGA. The current study is a re-analysis of a previous study that evaluated these adverse birth outcomes and exposure to VOC-contaminated drinking water at Camp Lejeune categorized simply as exposed versus unexposed [8]. Additionally, the previous study incorrectly categorized births before 1972 at HB as unexposed based on information available at the time. The current study used the results of extensive water modeling to estimate maternal exposures to mean monthly contaminant levels in the drinking water at Camp Lejeune which were unavailable during the previous study [1, 2]. We used the birth certificate data and housing data that were collected in the previous study, as well as the SGA norms, to evaluate these adverse birth outcomes; we did not collect any additional information on these births.

## Methods

This study received approval from the Centers for Disease Control and Prevention’s (CDC) Institutional Review Board.

### Study population

Since computerized birth certificates in North Carolina became available in 1968 and the contaminated wells on base were shut down in 1985, we included live singleton births 28–47 weeks gestation weighing ≥500 grams that occurred between 1968 and 1985 to mothers who lived at Camp Lejeune at delivery [8]. By cross referencing birth certificate data for Onslow County with Camp Lejeune housing records, we identified 11,896 births that met these criteria.

### Data collection

Outcomes of interest in this study were preterm birth and fetal growth retardation as measured by reduced MBW, TLBW, and SGA; data regarding these outcomes were obtained from birth certificates. Preterm births were defined as births occurring at less than 37 weeks of gestation. Gestational age was calculated using date of mother’s last menstrual period (LMP) from the birth certificate. TLBW was defined as full-term babies (≥37 weeks gestation) weighing <2,500 grams at birth. For SGA births, three categorizations were evaluated: births weighing <5^th^ and <10^th^ percentiles based on sex- and race-specific weight by gestational week norms from New Jersey and births weighing less <10^th^ percentile based on sex-specific growth curves for California [4, 9]. The New Jersey norms were determined using race- and sex-specific birth weights by gestational weeks for all singleton white and African American births in the state during 1985–1988. The California norms were based on sex-specific growth curves for white singleton births in the state from 1970–1976.

### Consent

Informed consent was not obtained from participants because this was a data-linkage study that did not involve contact with participants.

### Exposure assessment

To assign exposures, we used address information collected from birth certificates, base family housing records, and water modeling results. Each month of residence was linked to estimated levels of contaminants in drinking water serving that location. We examined the following time periods: each trimester and the entire pregnancy. For each time period examined, births were categorized as unexposed if mothers did not reside at Camp Lejeune, if their residence at Camp Lejeune received uncontaminated drinking water, or mothers were exposed for <1 week during that time period. A birth could be unexposed in the analysis of one trimester but categorized as exposed in the analysis of a different trimester. However if a birth was exposed in any trimester, then the birth was categorized as exposed in the analysis of the entire pregnancy.

Due to a lack of historical, contaminant-specific data, we conducted a historical reconstruction of contaminant levels in drinking water at Camp Lejeune. Modeling provided monthly average estimates of concentrations of contaminant-specific compounds in drinking water delivered to residences. The water modeling used extensive hydrogeological information as well as information on the sources of pollution, well pumping schedules, and the water distribution system of each of the treatment plants. Detailed information pertaining to the historical reconstruction was published in peer reviewed reports [1, 2].

### Data analysis

We used unconditional logistic regression in SAS 9.3 to individually compare the odds of preterm birth, TLBW, and SGA among the exposure categories [10]. We used linear regression in SAS 9.3 to compute MBW differences as indicated by the β coefficient. Reduced MBW among full-term babies was evaluated as a continuous variable by comparing birth weight differences by exposure categories. Unadjusted and adjusted odds ratios (ORs) and βs and their 95% confidence intervals (CIs) were calculated. We compared adjusted models to unadjusted models. In these comparisons, the unadjusted models only included births with complete data for the risk factor(s).

The following risk factors ascertained from birth certificates were evaluated for confounding: mother’s race, prenatal care, age of mother and father, parity, educational level of mother and father, sex of child, and if the mother had a previous fetal death. “Adequate” prenatal care was assigned based on the Kessner index, which uses start of prenatal care, number of prenatal visits, and duration of pregnancy to determine adequacy [11]. We also evaluated military rank (obtained from the family housing records) as a potential risk factor; rank was a surrogate measure of socio-economic status. If any potential risk factors were highly correlated, we evaluated the risk factor that was more strongly associated with the outcome. Each risk factor was included in a model with the exposure variable; if adjusted results differed from unadjusted results by >10%, the risk factor was selected as a potential confounder [12].

After all selected potential confounders were included in a model, a final model was determined using a backwards elimination process. Order of the elimination was determined by removing the potential confounder with the value closest to the null for the association between the confounder and the outcome and continuing until no factor could be removed without changing the estimate for the drinking water exposure by >10%. If there was no confounding by the risk factors, unadjusted models were presented.

We used two criteria to assess associations: (1) magnitude of the OR or β and (2) the exposure-response relationship, emphasizing monotonic trends in categorical exposure variables. A monotonic trend occurs when every change in the OR or MBW difference with increasing category of exposure is in the same direction, although the trend could have flat segments but never reverse direction [13]. Confidence intervals were only used to indicate the precision of the estimates [14–16]. We included p-values in tables for information purposes only. We did not use statistical significance testing to interpret findings [13, 15, 16].

For the primary analyses, exposure to each contaminant was evaluated separately. Exposure variables were categorized such that the reference group did not have residential exposure to the contaminant under evaluation (“unexposed”). For all contaminants except benzene, the exposed group was divided into four levels: < median value, ≥ median value, ≥75^th^ percentile, and ≥90^th^ percentile. Due to sparse data, those exposed to benzene were categorized into two levels: <1 part per billion (ppb) and ≥1 ppb. We analyzed average monthly concentration levels in the drinking water during each pregnancy trimester as well as during the entire pregnancy. Tables present results for average monthly concentration levels during the entire pregnancy. Trimester-specific results are provided in additional files (see Additional file 1: Table S1-S4). We mention in the text if results of specific trimesters differ from those of the entire pregnancy either because the magnitude of the association is different and/or there is an exposure-response relationship observed in a specific trimester that is not observed for the entire pregnancy.

Four types of secondary analyses were conducted. First, to obtain a visual characterization of the relationship between each outcome and average monthly concentration levels during each pregnancy trimester as well as during the entire pregnancy, we used a SAS macro to include a restricted cubic spline (RCS) function for the exposure as a continuous variable in the logistic and linear regression models [17]. Three knots were located at the 5th, 50th, and 95th percentiles of the average monthly exposure variable. (Because of sparse data, the knots for benzene could not be spaced symmetrically; instead, knots were located at the 10^th^, 75^th^ and 95^th^ percentiles.) The RCS function allowed the shape of the curve to vary within and between these knots and restricted the curve to be linear before the first knot and after the last knot. The resulting curve is useful for assessing whether the exposure-response relationship is adequately captured by the categorical exposure variables.

Second, to take into account correlations among births contributed by the same mother, we conducted generalized estimating equations (GEE) modeling using an exchangeable correlation structure. To identify mothers who contributed more than one singleton birth, it was necessary to match on mother’s name. However mother’s first name was missing for over one-third of the births, so it is likely that some mothers who contributed more than one birth were not identified. A total of 1,330 births (11.2% of births in the study) were identified among 646 mothers who contributed more than one singleton birth during the study period.

Third, when two contaminants were independently associated with an outcome, both contaminants were included in a model to determine which had the stronger association. Finally, analyses were conducted using those without residential exposure to any of the drinking water contaminants as a reference group.

## Results

A total of 11,896 births were identified and met the study inclusion criteria; all 11,896 births were included in analyses for SGA and preterm birth. We excluded 113 births with missing or incomplete information on LMP. Results are presented for SGA based on the 10^th^ percentile using sex- and race-specific weight by gestational week norms from New Jersey; we chose to present this categorization because results using all three SGA definitions were similar. The analyses of TLBW and reduced MBW were restricted to full-term infants (≥37 weeks gestation) and included 10,990 births. Results for the potential risk factors are shown in Tables 1 and 2. Information was missing for rank of the military member (n = 13, 1.1%), parity (n = 9, 0.8%), previous fetal death (n = 8, 0.7%), father’s age (n = 13, 1.1%), and father’s education (n = 13, 1.1%).

**Table 1:** **Potential risk factors for adverse pregnancy outcomes, Camp Lejeune, 1968–1985**

Risk factor	Small for gestational age (total births = 11,896)		Preterm birth (total births =11,896)		Term low birth weight (total births = 10,990)	
	#, %	OR	#, %	OR	#, %	OR
Race						
“white”	1072, 12.0	1.0 (ref.)	544, 6.1	1.0 (ref.)	155, 1.9	1.0 (ref.)
“other”	502, 16.9	1.5 (1.3,1.7)	362, 12.2	2.1 (1.9, 2.5)	91, 3.5	1.9 (1.5, 2.5)
Prenatal care*						
Adequate	624, 11.9	1.0 (ref.)	258, 4.9	1.0 (ref.)	76, 1.5	1.0 (ref.)
Inadequate	950, 14.2	1.2 (1.1, 1.4)	648, 9.7	2.1 (1.8, 2.4)	170, 2.8	1.9 (1.4, 2.5)
Rank^**†**^						
Officer	166, 10.1	1.0 (ref.)	62, 3.8	1.0 (ref.)	25, 1.6	1.0 (ref.)
Enlisted	1408, 13.7	1.4 (1.2, 1.7)	844, 8.2	2.3 (1.8, 3.0)	219, 2.3	1.5 (1.0, 2.2)
Mother’s age						
<20	304, 15.5	1.2 (1.1, 1.7)	196, 10.0	1.4 (1.2, 1.7)	42, 2.4	1.1 (0.8, 1.5)
20-29	1132, 12.9	1.0 (ref.)	623, 7.1	1.0 (ref.)	176, 2.2	1.0 (ref.)
30-34	108, 11.8	0.9 (0.7, 1.1)	67, 7.3	1.0 (0.8, 1.3)	21, 2.5	1.1 (0.7, 1.8)
≥35	30, 12.9	1.0 (0.7, 1.5)	20, 8.6	1.2 (0.8, 2.0)	7, 3.3	1.5 (0.7, 3.3)
Father’s age						
<20	100, 15.9	1.2 (1.0, 1.5)	62, 9.8	1.3 (1.0, 1.7)	13, 2.3	1.0 (0.6, 1.8)
20-29	1284, 13.4	1.0 (ref.)	733, 7.6	1.0 (ref.)	195, 2.2	1.0 (ref.)
30-34	128, 10.7	0.8 (0.6, 0.9)	75, 6.3	0.8 (0.6, 1.0)	25, 2.2	1.0 (0.7, 1.5)
≥35	62, 13.4	1.0 (0.8, 1.3)	36, 7.8	1.0 (0.7, 1.5)	13, 3.1	1.4 (0.8, 2.5)
Parity						
Multiparous	953, 12.2	1.0 (ref.)	650, 8.3	1.0 (ref.)	168, 2.4	1.0 (ref.)
Primiparous	619, 15.2	1.3 (1.2, 1.4)	254, 6.2	0.7 (0.6, 0.9)	77, 2.0	0.9 (0.7, 1.1)
Mom’s education						
Not a college graduate	1474, 13.5	1.0 (ref.)	859, 7.9	1.0 (ref.)	231, 2.3	1.0 (ref.)
College grad	100, 10.6	0.8 (0.6, 0.9)	46, 4.9	0.6 (0.4, 0.8)	15, 1.7	0.7 (0.4-1.2)
Dad’s education						
Not a college graduate	1410, 13.6	1.0 (ref.)	844, 8.1	1.0 (ref.)	223, 2.3	1.0 (ref.)
College graduate	163, 11.0	0.8 (0.7, 0.9)	59, 4.0	0.5 (0.4, 0.6)	23, 1.6	0.7 (0.4-1.1)
Sex of child						
Female	760, 12.9	1.0 (ref.)	419, 7.1	1.0 (ref.)	147, 2.7	1.0 (ref.)
Male	814, 13.6	1.1 (1.0, 1.2)	487, 8.1	1.2 (1.0, 1.3)	99, 1.8	0.7 (0.5, 0.9)
Previous fetal death						
0	1287, 13.0	1.0 (ref.)	719, 7.2	1.0 (ref.)	194, 2.1	1.0 (ref.)
≥ 1	285, 14.6	1.1 (1.0, 1.3)	185, 9.5	1.3 (1.1, 1.6)	51, 2.9	1.4 (1.0, 1.9)

* “Adequate” if care began during 1^st^ trimester and sufficient number of visits given gestational age of child at birth (Kessner 1973) [11]; ^**†**^rank of military member.

**Table 2:** **Potential risk factors for mean birth weight difference, Camp Lejeune, 1968–1985**

Risk factor	Mean birth weight (total births = 10,990)	
	#, %	MBW difference (grams)
Race		
“white”	8388, 76.3	Reference
“other”	2602, 23.7	−170.6 (−191.3, −149.8)
Prenatal care*		
Adequate	4985, 45.4	Reference
Inadequate	6003, 54.6	−71.1 (−89.0, −53.2)
Rank^**†**^		
Officer	1578, 14.4	Reference
Enlisted	9412, 85.6	−92.4 (−117.7, −67.0)
Mother’s age		
<20	1772, 16.1	−57.5 (−81.9, −33.0)
20-29	8155, 74.2	Reference
30-34	850, 7.7	71.9 (38.3, 105.6)
≥35	213, 1.9	68.7 (3.9, 133.5)
Father’s age		
<20	557, 5.2	−22.2 (−63.0, 18.6)
20-29	8874, 80.8	Reference
30-34	1121, 10.2	80.7 (51.1, 110.3)
≥35	426, 3.9	58.9 (12.6, 105.3)
Parity		
Multiparous	7161, 65.2	Reference
Primiparous	3822, 34.8	−69.9 (−88.6, −51.2)
Mom’s education		
Not a college graduate	10090, 91.8	Reference
College grad	896, 8.2	74.4 (41.8, 106.9)
Dad’s education		
Not a college graduate	9559, 87.1	Reference
College graduate	1421, 12.9	81.8 (55.3, 108.4)
Sex of child		
Female	5470, 49.8	Reference
Male	5520, 50.2	145.9 (128.2, 163.5)
Previous fetal death		
0	9216, 83.9	Reference
≥ 1	1768, 16.1	−6.6 (−30.5, 18.1)

* “Adequate” if care began during 1^st^ trimester and sufficient number of visits given gestational age of child at birth (Kessner 1973); ^**†**^rank of military member.

The results for the degradation products, DCE and vinyl chloride, are not presented because these chemicals were highly correlated with PCE (Kendall’s tau for PCE and vinyl chloride = 0.95 and Kendall’s tau for PCE and DCE = 0.95). Results for PCE, TCE, and benzene are presented.

For SGA, the OR for TCE in the highest exposure category during the entire pregnancy was 1.5 (95% CI: 1.2, 1.9), but we did not observe a monotonic exposure-response relationship (Table 3). The spline for TCE and SGA indicated increasing ORs with increasing exposure up to about the 95^th^ percentile of exposure and then a slight tailing off at higher exposures (See Additional file 2: Figure S1). ORs for SGA and the highest PCE and benzene exposure categories during the entire pregnancy were 1.0 (95% CI: 0.8, 1.2) and 1.2 (95% CI: 0.9, 1.5), respectively, with no monotonic exposure-response relationships.

**Table 3:** **Small for gestational age and average VOC exposure, entire pregnancy, Camp Lejeune, 1968-1985**

Exposure	Small for gestation age		OR (95% CI)	p value
	No #, %	Yes #, %		
**Tetrachloroethylene (PCE)**				
No exposure	3246, 31.4	476, 30.2	1.0 (ref.)	
>0- < 35.8 ppb	3558, 34.5	531, 33.7	1.0 (0.9-1.2)	0.80
≥35.8- < 52.7 ppb	1730, 16.8	309, 19.6	1.2 (1.0-1.4)	0.01
≥52.7- < 81.4 ppb	1075, 10.4	154, 9.8	1.0 (0.8-1.2)	0.81
≥ 81.4 ppb	713, 6.9	104, 6.6	1.0 (0.8-1.2)	0.96
**Trichloroethylene (TCE)**				
No exposure	1891, 18.3	238, 15.1	1.0 (ref.)	
>0- < 1.7 ppb	4306, 41.7	676, 42.9	1.2 (1.1-1.5)	0.01
≥1.7- < 3.2 ppb	2057, 19.9	326, 20.7	1.3 (1.1-1.5)	0.01
≥3.2- < 9.8 ppb	1248, 12.1	180, 11.4	1.1 (1.0-1.4)	0.20
≥ 9.8 ppb	820, 7.9	154, 9.8	1.5 (1.2-1.9)	<0.01
**Benzene**				
No exposure	7356, 71.3	1107, 70.3	1.0 (ref.)	
>0- < 1 ppb	2335, 22.6	356, 22.6	1.0 (0.9-1.2)	0.84
≥ 1 ppb	631, 6.1	111, 7.1	1.2 (0.9-1.5)	0.15

For preterm birth, after adjusting for mother’s race, the OR for 2^nd^ trimester exposure to the highest category of PCE was 1.5 (95% CI: 1.1, 2.0) compared with an OR of 1.3 (95% CI: 1.0, 1.6) for the highest PCE exposure category during the entire pregnancy (Table 4, Additional file 1: Table S2A). The spline for 2^nd^ trimester PCE exposure and preterm birth indicated a monotonic relationship (see Additional file 2: Figure S2). ORs for preterm birth and the highest TCE and benzene exposure categories during the entire pregnancy were 1.1 and 0.8, respectively.

**Table 4:** **Preterm birth and average VOC exposure, entire pregnancy, Camp Lejeune, 1968-1985**

Exposure	Preterm birth		OR* (95% CI)	p value
	No #, %	Yes #, %		
**Tetrachloroethylene (PCE)**				
No exposure	3453, 31.4	269, 29.7	1.0 (ref.)	
>0- < 35.8 ppb	3763, 34.2	326, 36.0	1.0 (0.9-1.2)	0.67
≥35.8- < 52.7 ppb	1895, 17.2	144, 15.9	0.9 (0.7-1.1)	0.32
≥52.7- < 81.4 ppb	1148, 10.4	81, 8.9	0.8 (0.6-1.0)	0.09
≥ 81.4 ppb	731, 6.7	86, 9.5	1.3 (1.0-1.6)	0.08
**Trichloroethylene (TCE)**				
No exposure	1979, 18.0	150, 16.6	1.0 (ref.)	
>0- < 1.7 ppb	4599, 41.8	383, 42.3	1.1 (0.9-1.3)	0.35
≥1.7- < 3.2 ppb	2209, 20.1	174, 19.2	1.0 (0.8-1.3)	0.74
≥3.2- < 9.8 ppb	1304, 11.9	124, 13.7	1.3 (1.0-1.6)	0.07
≥ 9.8 ppb	899, 8.2	75, 8.3	1.1 (0.8-1.5)	0.51
**Benzene**				
No exposure	7820, 71.2	643, 71.0	1.0 (ref.)	
>0- < 1.0 ppb	2474, 22.5	217, 24.0	1.1 (0.9-1.3)	0.43
≥ 1.0 ppb	696, 6.3	46, 5.0	0.8 (0.6-1.1)	0.17

*PCE was adjusted for mother’s race.

For TLBW, the OR for TCE in the highest exposure category during the entire pregnancy was 1.3 (95% CI: 0.8, 2.2) (Table 5). Additionally, the OR for TLBW and 2^nd^ trimester exposure to the highest category of TCE was 1.6 (95% CI: 1.0, 2.6) and we observed a monotonic exposure-response relationship (ORs for the other 2^nd^ trimester TCE categorizations were 1.3, 1.3, and 1.5) (see Additional file 1: Table S3B). The spline for 2^nd^ trimester TCE exposure and TLBW indicated a non-monotonic relationship with ORs rising to 1.8 and then falling below 1.5 for exposures above the 97.5 percentile (See Additional file 2: Figure S3). The OR for exposure to the highest category of benzene during the entire pregnancy was 1.5 (95% CI: 0.9, 2.3), and there was a monotonic exposure-response relationship. The spline also indicated a monotonic relationship (See Additional file 2: Figure S4). The OR for TLBW and the highest PCE exposure category during the entire pregnancy was <1.0.

**Table 5:** **Term low birth weight and average VOC exposure, entire pregnancy, Camp Lejeune, 1968-1985**

Exposure	Term low birth weight		OR (95% CI)	p value
	No #, %	Yes #, %		
**Tetrachloroethylene (PCE)**				
No exposure	3376, 31.4	77, 31.3	1.0 (ref.)	
>0 - <35.9 ppb	3689, 34.3	80, 32.5	1.0 (0.7- 1.3)	0.75
≥35.9- <52.6 ppb	1834, 17.1	48, 19.5	1.1 (0.9-1.9)	0.46
≥52.6 - <80.7 ppb	1102, 10.3	29, 11.8	1.2 (0.7-1.7)	0.52
≥80.7 ppb	743, 6.9	12, 4.9	0.7 (0.5-1.4)	0.27
**Trichloroethylene (TCE)**				
No exposure	1938, 18.0	41, 16.7	1.0 (ref.)	
>0 - <1.7 ppb	4502, 41.9	97, 39.4	1.0 (0.7-1.5)	0.92
≥1.7- <3.1 ppb	2045, 19.0	57, 23.1	1.3 (0.9-2.0)	0.18
≥3.1 - <9.8 ppb	1384, 12.9	27, 11.0	0.9 (0.6-1.5)	0.75
≥9.8 ppb	875, 8.1	24, 9.8	1.3 (0.8-2.2)	0.32
**Benzene**				
No exposure	7651, 71.2	169, 68.7	1.0 (ref.)	
>0- < 1 ppb	2419, 22.5	55, 22.4	1.0 (0.8-1.4)	0.85
≥ 1 ppb	674, 6.3	22, 8.9	1.5 (0.9-2.3)	0.09

For MBW, after adjusting the TCE model for sex of child, mother’s race, and parity, we observed a reduced MBW of −78.3 g (95% CI: −115.0, −41.7; p-value <0.01) in the highest exposure category during the entire pregnancy (Table 6). Adjusted results for 3^rd^ trimester TCE exposure resulted in a reduced MBW of −92.9 g (95% CI: −129.4, −56.5; p-value <0.01) in the highest exposure category (see Additional file 1: Table S4B). The splines for TCE exposure over the entire pregnancy and over the 3^rd^ trimester were similar and indicated a leveling off in the decline in mean birth weight by the 95^th^ percentile of exposure (See Additional file 2: Figure S5). After adjusting the benzene model for prenatal care, sex of child, mother’s race, parity, and rank of the military member, we observed a reduced MBW of −36.2 g (95% CI: −72.3, −0.1; p-value =0.05) for exposure during the entire pregnancy, and there was a monotonic exposure-response relationship. Adjusted results for 2^nd^ trimester exposure to benzene resulted in a reduced MBW of −47.4 g (95% CI: −81.2, −13.7), and there was a monotonic exposure-response relationship (see Additional file 1: Table S4C). Adjusted results for PCE exposure during the entire pregnancy did not indicate a decrease in MBW. However, 1^st^ trimester exposure to PCE resulted in a slight MBW decrease for the highest category of exposure (−10.4 g, 95% CI: −55.5, 34.7) (see Additional file 1: Table S4A).

**Table 6:** **Birth weight and average VOC exposure during pregnancy, term births, Camp Lejeune 1968-1985**

Exposure	#	Mean birth weight difference in grams (95% CI)	p value
Tetrachloroethylene (PCE)*			
Unexposed (ref)	3453	--	
>0 - <35.9 ppb	3769	4.4 (−17.4, 26.1)	0.69
≥35.9- <52.6 ppb	1882	−28.5 (−55.1, −1.9)	0.04
≥52.6 - <80.7 ppb	1131	3.8 (−28.3, 36.0)	0.82
≥80.7 ppb	755	8.2 (−29.5, 46.0)	0.67
**Trichloroethylene (TCE)** ^**†**^			
Unexposed (ref)	1979	--	
>0 - <1.7 ppb	4599	−42.2 (−66.7, −17.7)	<0.01
≥1.7- <3.1 ppb	2102	−48.9 (−77.4, −20.3)	<0.01
≥3.1 - <9.8 ppb	1411	−40.5 (−72.3, −8.7)	0.01
≥9.8 ppb	899	−78.3 (−115.0, −41.7)	<0.01
**Benzene§**			
Unexposed (ref)	7820	--	
>0 - <1 ppb	2474	−14.5 (−35.5, 6.5)	0.18
≥1 ppb	696	−36.2 (−72.3, −0.1)	0.05

*Adjusted for prenatal care, sex of child, mother’s race, mother’s age, mother’s education, parity, mother had a previous fetal death, father’s age, and rank of military member.

^**†**^Adjusted for sex of child, mother’s race, and parity.

^**§**^Adjusted for prenatal care, sex of child, mother’s race, parity, and rank of military member.

The categorical results indicated associations between TCE and SGA, TLBW and MBW, PCE and preterm birth, and benzene and TLBW and MBW. Since both TCE and benzene were associated with TLBW and reduced MBW, we included both contaminants in models for these two outcomes. We modeled 2^nd^ trimester exposures for TLBW because the ORs for TCE were higher in this trimester compared with the entire pregnancy. For TLBW, both contaminants remained associated although their ORs at the high exposure categories were slightly reduced (TCE OR =1.2, 95% CI: 0.6, 2.7 and benzene OR =1.4, 95% CI: 0.6, 3.0). However, for reduced MBW modeled for the entire pregnancy, benzene no longer was associated (31.9, 95% CI: −35.9, 99.7) and the MBW deficit for TCE at the high exposure level increased to −98.0 g (95% CI: −162.1, −33.9).

The GEE results indicated that the analyses were not affected by correlated births from the same mother. Results obtained in the secondary analyses using an unexposed group consisting of those without exposure to any drinking water contaminants were not presented because they were similar to results obtained in the primary analyses in most instances or produced only a small increase in ORs.

## Discussion

We were able to study the relationship between adverse pregnancy outcomes and contaminated drinking water among a large number of births. Computer modeling of the drinking water system at Camp Lejeune during 1968–1985 provided ATSDR with extensive estimates of the exposure [1, 2].

We observed ORs of 1.5 and 1.3 for SGA and TLBW, respectively, and a reduced MBW of −78.3 g for the highest exposure category to TCE during the entire pregnancy. Additionally, the OR for 2^nd^ trimester exposure to the highest TCE category and TLBW was 1.6, and we observed a monotonic exposure-response relationship. Exposure to TCE did not increase risk for preterm birth (OR for the highest exposure category to TCE during the entire pregnancy was 1.1). The SGA finding is consistent with a study in Woburn, MA (OR = 1.6) and the preterm birth finding is consistent with studies in Woburn, MA (OR ≤1.0) and northern New Jersey (OR = 1.0) [4–6]. A Finnish study found an adjusted OR of 1.26 for low birth weight and occupational solvent exposure in the 2^nd^ trimester [18]. A study of trimester-specific exposures to air pollutants also found the strongest risk in the 2^nd^ trimester for TLBW and preterm birth (adjusted ORs for both outcomes were 1.139 for particulate matter with aerodynamic diameter <2.5 micrometers [19].

For PCE, we observed adjusted ORs of 1.3 and 1.5 for preterm birth and the highest exposure category during the entire pregnancy and 2^nd^ trimester, respectively. Exposure to PCE did not increase risk for SGA or TLBW (ORs for the highest exposure categories during the entire pregnancy were ≤1.0), or produce MBW reductions (β for the highest exposure category during the entire pregnancy was 8.2 g). The MBW finding is consistent with a previous study which found an adjusted mean difference in birth weight of 15.2 g for mothers exposed to the highest quartile of PCE around the time of conception [3].

We observed monotonic exposure-response relationships for benzene exposure during the entire pregnancy and TLBW (highest category OR = 1.5) and adjusted MBW difference (highest category β = −37.1 g). However, when TCE was added to the model with MBW, the association with benzene disappeared. Exposure to benzene did not increase risk for SGA or preterm birth (ORs for the highest exposure categories during the entire pregnancy were ≤1.2). We are unaware of any previous studies linking drinking water exposures to benzene and TLBW or reduced MBW.

In this study, mother’s race was a confounder for preterm birth and exposure to PCE and for reduced MBW and exposure to the contaminants. SGA births were based on sex- and race-specific weight by gestational week norms so we did not further adjust for mother’s race. Parity and prenatal care, along with sex of the child; mother’s race, age and education; mother had a previous fetal death; father’s age; and rank of the military member confounded the relationship between MBW and the contaminants, but were adjusted as needed in our final model.

Smoking during pregnancy is a known risk factor for adverse birth outcomes such as low birth weight, preterm birth, and SGA [20–23]. We could not control for smoking in this study because smoking was not recorded on birth certificates during the study period. However, we explored the effect that smoking may have on our results by conducting a quantitative bias sensitivity analysis to determine how large a difference in smoking status would be needed to change the OR for the highest exposure to TCE and SGA by >10%. Assuming an OR between smoking and SGA of 2.6 and that 30% of unexposed mothers smoked [21], the prevalence of smoking among mothers in the highly exposed TCE group would have to be >40% to create a >10% change in the OR. However, we expect the smoking status among pregnant women at Camp Lejeune to vary very little based on drinking water exposure status.

### Limitations

This study relied on vital statistics data and Camp Lejeune housing records and only included births occurring in women who lived on base at the time of delivery. We were unable to include births to women who were pregnant while living at Camp Lejeune but who delivered off-base. We did not conduct interviews to obtain more detailed information on residential history or other maternal characteristics (e.g., alcohol consumption, weight gain during pregnancy, smoking status) not captured by birth certificates during the study period. However, in order for any risk factor to have a confounding impact on the findings, it needs to be strongly associated with the exposure. It is unknown how unmeasured confounding might affect the results of this study. For TLBW, the confidence intervals were wider than the other outcomes because of small numbers in the highest exposure category.

We only modeled residential exposures to drinking water contaminants. Since drinking water exposures could occur during daily activities all over the base, some mothers categorized as unexposed may have had some drinking water exposure. This exposure misclassification bias could have distorted exposure-response trends in comparisons involving more than two levels. Therefore, although we emphasize monotonic response curves, we do not ignore results when the exposure-response is not monotonic.

## Conclusion

Findings suggested associations between in utero exposure to TCE and SGA, TLBW and reduced MBW; in utero exposure to benzene and TLBW; and in utero exposure to PCE and preterm birth. For TLBW, we observed a monotonic exposure-response relationship for TCE exposure during the 2^nd^ trimester and for benzene exposure during the entire pregnancy. For PCE and preterm birth, the strongest association was observed for 2^nd^ trimester exposures. The study found no evidence suggesting any other associations between outcomes and exposures. Results of this study add to the scientific literature on the health effects of exposures to these chemicals in drinking water.

## Electronic supplementary material

Additional file 1: Table S1: **a**. Small for gestational age and average PCE exposure, by trimesters, Camp Lejeune, 1968-1985 **b**. Small for gestational age and average TCE exposure, by trimesters, Camp Lejeune, 1968-1985 **c**. Small for gestational age and average benzene exposure, by trimesters, Camp Lejeune, 1968-1985. **Table S2 a**. Preterm birth and average PCE exposure, by trimesters, Camp Lejeune, 1968-1985 **b**. Preterm birth and average TCE exposure, by trimesters, Camp Lejeune, 1968-1985 **c**. Preterm birth and average benzene exposure, by trimesters, Camp Lejeune, 1968-1985. **Table S3 a**. Term low birth weight and average PCE exposure, by trimesters, Camp Lejeune, 1968-1985 **b**. Term low birth weight and average TCE exposure, by trimesters, Camp Lejeune, 1968-1985 **c**. Term low birth weight and average benzene exposure, by trimesters, Camp Lejeune, 1968-1985. **Table S4 a**. Birth weight and average PCE exposure, by trimesters, term births, Camp Lejeune, 1968-1985 **b**. Birth weight and average TCE exposure, by trimesters, term births, Camp Lejeune, 1968-1985 **c**. Birth weight and average benzene exposure, by trimesters, term births, Camp Lejeune, 1968-1985. (DOCX 57 KB)

Additional file 2: Figures S1-S5: Splines of selected outcomes and exposures. (DOCX 108 KB)

## Abbreviations

DCE: 1,2-dichloroethylene
ATSDR: Agency for Toxic Substances and Disease Registry
CI: Confidence interval
GEE: Generalized estimating equations
HP: Hadnot Point
HB: Holcomb Boulevard
LMP: Last menstrual period
MCL: Maximum contaminant level
MBW: Mean birth weight
OR: Odds ratio
ppb: Parts per billion
PHA: Public health assessment
RCS: Restricted cubic spline
SGA: Small for gestational age
TT: Tarawa Terrace
TLBW: Term low birth weight
PCE: Tetrachloroethylene
TCE: Trichloroethylene
USMC: United States Marine Corps
VOCs: Volatile organic compounds
EPA: United States Environmental Protection Agency.
