# Supplementary material for: Evaluation of contaminated drinking water and preterm birth, small for gestational age, and birth weight at Marine Corps Base Camp Lejeune, North Carolina: a cross-sectional study
Source: Environ Health. 2014 Nov 20;13:99. doi: 10.1186/1476-069X-13-99 (PMC4247681; doi:10.1186/1476-069X-13-99)
Supplement: Supplementary file 1 — Additional file 1: Table S1: a. Small for gestational age and average PCE exposure, by trimesters, Camp Lejeune, 1968-1985 b. Small for gestational age and average TCE exposure, by trimesters, Camp Lejeune, 1968-1985 c. Small for gestational age and average benzene exposure, by trimesters, Camp Lejeune, 1968-1985. Table S2 a. Preterm birth and average PCE exposure, by trimesters, Camp Lejeune, 1968-1985 b. Preterm birth and average TCE exposure, by trimesters, Camp Lejeune, 1968-1985 c. Preterm birth and average benzene exposure, by trimesters, Camp Lejeune, 1968-1985. Table S3 a. Term low birth weight and average PCE exposure, by trimesters, Camp Lejeune, 1968-1985 b. Term low birth weight and average TCE exposure, by trimesters, Camp Lejeune, 1968-1985 c. Term low birth weight and average benzene exposure, by trimesters, Camp Lejeune, 1968-1985. Table S4 a. Birth weight and average PCE exposure, by trimesters, term births, Camp Lejeune, 1968-1985 b. Birth weight and average TCE exposure, by trimesters, term births, Camp Lejeune, 1968-1985 c. Birth weight and average benzene exposure, by trimesters, term births, Camp Lejeune, 1968-1985. (DOCX 57 KB) [file 12940_2014_799_MOESM1_ESM.docx]

**Additional files – Tables S1-4**

**Evaluation of contaminated drinking water and preterm birth, small for gestational age, and birth weight at Marine Corps Base Camp Lejeune, North Carolina: A cross-sectional study**

Perri Zeitz Ruckart, Frank J. Bove, Morris Maslia

**Additional file 1A. Small for gestational age and average PCE exposure, by trimesters, Camp Lejeune, 1968-1985**

| **Exposure** | **Small for gestational age** | | **OR (95% CI)** | **p value** |
| --- | --- | --- | --- | --- |
|  | **No**  **#** | **Yes #** |  |  |
| **First trimester** |  |  |  |  |
| No exposure | 6114 | 915 | 1.0 (ref.) |  |
| >0-<44.4 ppb | 2085 | 346 | 1.1 (1.0-1.3) | 0.13 |
| ≥44.4-<69.4 ppb | 1055 | 170 | 1.1 (0.9-1.3) | 0.41 |
| ≥69.4-<93.6 ppb | 642 | 81 | 0.8 (0.7-1.1) | 0.17 |
| ≥ 93.6 ppb | 426 | 62 | 1.0 (0.7-1.3) | 0.84 |
| **Second trimester** |  |  |  |  |
| No exposure | 4971 | 715 | 1.0 (ref.) |  |
| >0-<44.8 ppb | 2679 | 433 | 1.1 (1.0-1.3) | 0.07 |
| ≥44.8-<78.7 ppb | 1319 | 227 | 1.2 (1.0-1.4) | 0.03 |
| ≥78.7-<95.3 ppb | 805 | 125 | 1.1 (0.9-1.3) | 0.46 |
| ≥ 95.3 ppb | 548 | 74 | 0.9 (0.7-1.2) | 0.63 |
| **Third trimester** |  |  |  |  |
| No exposure | 4055 | 586 | 1.0 (ref.) |  |
| >0-<44.1 ppb | 3131 | 512 | 1.1 (1.0-1.3) | 0.06 |
| ≥44.1-<68.8 ppb | 1548 | 249 | 1.1 (0.9-1.3) | 0.19 |
| ≥68.8-<93.2 ppb | 943 | 134 | 1.0 (0.8-1.2) | 0.87 |
| ≥ 93.2 ppb | 645 | 93 | 1.0 (0.8-1.3) | 0.98 |

**Additional file 1B. Small for gestational age and average TCE exposure, by trimesters, Camp Lejeune, 1968-1985**

| **Exposure** | **Small for gestational age** | | **OR (95% CI)** | **p value** |
| --- | --- | --- | --- | --- |
|  | **No**  **#** | **Yes #** |  |  |
| **First trimester** |  |  |  |  |
| No exposure | 5343 | 784 | 1.0 (ref.) |  |
| >0-<2.0 ppb | 2510 | 419 | 1.1 (1.0-1.3) | 0.05 |
| ≥2.0-<3.9 ppb | 1280 | 175 | 0.9 (0.8-1.1) | 0.43 |
| ≥3.9-<15.8 ppb | 703 | 103 | 1.0 (0.8-1.2) | 0.99 |
| ≥ 15.8 ppb | 486 | 93 | 1.3 (1.0-1.6) | 0.03 |
| **Second trimester** |  |  |  |  |
| No exposure | 3972 | 525 | 1.0 (ref.) |  |
| >0-<2.3 ppb | 3139 | 530 | 1.3 (1.1-1.5) | <0.01 |
| ≥2.3-<3.9 ppb | 1601 | 244 | 1.2 (1.0-1.4) | 0.09 |
| ≥3.9-<20.3 ppb | 984 | 161 | 1.2 (1.0-1.5) | 0.03 |
| ≥ 20.3 ppb | 626 | 114 | 1.4 (1.1-1.7) | <0.01 |
| **Third trimester** |  |  |  |  |
| No exposure | 2860 | 371 | 1.0 (ref.) |  |
| 3736 | 3736 | 624 | 1.3 (1.1-1.5) | <0.01 |
| ≥2.1-<3.9 ppb | 1953 | 277 | 1.1 (0.9-1.3) | 0.29 |
| ≥3.9-<16.8 ppb | 1049 | 169 | 1.2 (1.0-1.5) | 0.03 |
| ≥ 16.8 ppb | 724 | 133 | 1.4 (1.1-1.8) | <0.01 |

**Additional file 1C. Small for gestational age and average benzene exposure, by trimesters, Camp Lejeune, 1968-1985**

| **Exposure** | **Small for gestational age** | | **OR (95% CI)** | **p value** |
| --- | --- | --- | --- | --- |
|  | **No**  **#** | **Yes #** |  |  |
| **First trimester** |  |  |  |  |
| No exposure | 8906 | 1351 | 1.0 (ref.) |  |
| >0-<1 ppb | 938 | 140 | 1.0 (0.8-1.2) | 0.86 |
| ≥ 1 ppb | 478 | 83 | 1.1 (0.9-1.5) | 0.27 |
| **Second trimester** |  |  |  |  |
| No exposure | 8596 | 1278 | 1.0 (ref.) |  |
| >0-<1 ppb | 996 | 159 | 1.1 (0.9-1.3) | 0.43 |
| ≥ 1 ppb | 730 | 137 | 1.3 (1.0-1.6) | 0.02 |
| **Third trimester** |  |  |  |  |
| No exposure | 8286 | 1240 | 1.0 (ref.) |  |
| >0-<1 ppb | 1226 | 186 | 1.0 (0.8-1.2) | 0.87 |
| ≥ 1 ppb | 810 | 148 | 1.2 (1.0-1.5) | 0.03 |

**Additional file 2A. Preterm birth and average PCE exposure*, by trimesters, Camp Lejeune, 1968-1985**

| **Exposure** | **Preterm birth** | | **OR (95% CI)** | **p value** |
| --- | --- | --- | --- | --- |
|  | **No**  **#** | **Yes #** |  |  |
| **First trimester** |  |  |  |  |
| No exposure | 6529 | 500 | 1.0 (ref.) |  |
| >0-<44.4 ppb | 2222 | 209 | 1.2 (1.0-1.4) | 0.08 |
| ≥44.4-<69.4 ppb | 1135 | 90 | 1.0 (0.8-1.2) | 0.79 |
| ≥69.4-<93.6 ppb | 658 | 65 | 1.1 (0.9-1.5) | 0.37 |
| ≥ 93.6 ppb | 446 | 42 | 1.1 (0.8-1.5) | 0.71 |
| **Second trimester** |  |  |  |  |
| No exposure | 5299 | 387 | 1.0 (ref.) |  |
| >0-<44.8 ppb | 2858 | 254 | 1.2 (1.0-1.4) | 0.08 |
| ≥44.8-<78.7 ppb | 1422 | 124 | 1.1 (0.9-1.4) | 0.27 |
| ≥78.7-<95.3 ppb | 858 | 72 | 1.0 (0.8-1.3) | 0.84 |
| ≥ 95.3 ppb | 553 | 69 | 1.5 (1.1-2.0) | <0.01 |
| **Third trimester** |  |  |  |  |
| No exposure | 4285 | 356 | 1.0 (ref.) |  |
| >0-<44.1 ppb | 3381 | 262 | 0.9 (0.7-1.0) | 0.15 |
| ≥44.1 -<68.8 ppb | 1634 | 163 | 1.1 (0.9-1.3) | 0.33 |
| ≥68.8-<93.2 ppb | 1009 | 68 | 0.7 (0.5-0.9) | 0.02 |
| ≥ 93.2 ppb | 681 | 57 | 0.9 (0.7-1.2) | 0.37 |

*adjusted for mother’s race

**Additional file 2B. Preterm birth and average TCE exposure, by trimesters, Camp Lejeune, 1968-1985**

| **Exposure** | **Preterm birth** | | **OR (95% CI)** | **p value** |
| --- | --- | --- | --- | --- |
|  | **No**  **#** | **Yes #** |  |  |
| **First trimester** |  |  |  |  |
| No exposure | 5700 | 427 | 1.0 (ref.) |  |
| >0-<2.0 ppb | 2691 | 238 | 1.2 (1.0-1.4) | 0.05 |
| ≥2.0-<3.9 ppb | 1326 | 129 | 1.3 (1.1-1.6) | 0.01 |
| ≥3.9-<15.8 ppb | 734 | 72 | 1.3 (1.0-1.7) | 0.04 |
| ≥ 15.8 ppb | 539 | 40 | 1.0 (0.7-1.4) |  |
| **Second trimester** |  |  |  |  |
| No exposure | 4212 | 285 | 1.0 (ref.) |  |
| >0-<2.3 ppb | 3376 | 293 | 1.3 (1.1-1.5) | <0.01 |
| ≥2.3-<3.9 ppb | 1693 | 152 | 1.3 (1.1-1.6) | <0.01 |
| ≥3.9-<20.3 ppb | 1025 | 120 | 1.7 (1.4-2.2) | <0.01 |
| ≥ 20.3 ppb | 684 | 56 | 1.2 (0.9-1.6) | 0.21 |
| **Third trimester** |  |  |  |  |
| No exposure | 2977 | 254 | 1.0 (ref.) |  |
| >0-<2.1 ppb | 4045 | 315 | 0.9 (0.8-1.1) | 0.30 |
| ≥2.1-<3.9 ppb | 2044 | 186 | 1.1 (0.9-1.3) | 0.52 |
| ≥3.9-16.8 ppb | 1130 | 88 | 0.9 (0.7-1.2) | 0.48 |
| ≥ 16.8 ppb | 794 | 63 | 0.9 (0.7-1.2) | 0.62 |

**Additional file 2C. Preterm birth and average benzene exposure, by trimesters, Camp Lejeune, 1968-1985**

| **Exposure** | **Preterm birth** | | **OR (95% CI)** | **p value** |
| --- | --- | --- | --- | --- |
|  | **No**  **#** | **Yes #** |  |  |
| **First trimester** |  |  |  |  |
| No exposure | 9492 | 765 | 1.0 (ref.) |  |
| >0-<1 ppb | 976 | 102 | 1.3 (1.0-1.6) | 0.02 |
| ≥ 1 ppb | 522 | 39 | 0.9 (0.7-1.3) | 0.66 |
| **Second trimester** |  |  |  |  |
| No exposure | 9141 | 733 | 1.0 (ref.) |  |
| >0-<1 ppb | 1051 | 104 | 1.2 (1.0-1.5) | 0.06 |
| ≥ 1 ppb | 798 | 69 | 1.1 (0.8-1.4) | 0.57 |
| **Third trimester** |  |  |  |  |
| No exposure | 8788 | 738 | 1.0 (ref.) |  |
| >0-<1 ppb | 1306 | 106 | 1.0 (0.8-1.2) | 0.75 |
| ≥ 1 ppb | 896 | 62 | 0.8 (0.6-1.1) | 0.16 |

**Additional file 3A. Term low birth weight and average PCE exposure, by trimesters, Camp Lejeune, 1968-1985**

| **Exposure** | **Term low birth weight** | | **OR (95% CI)** | **p value** |
| --- | --- | --- | --- | --- |
|  | **No**  **#** | **Yes #** |  |  |
| **First trimester** |  |  |  |  |
| No exposure | 6391 | 138 | 1.0 (ref.) |  |
| >0-<44.4 ppb | 2162 | 60 | 1.3 (0.9-1.7) | 0.11 |
| ≥44.4-<69.3 ppb | 1092 | 31 | 1.3 (0.9-2.0) | 0.17 |
| ≥69.3-<92.9 ppb | 660 | 10 | 0.7 (0.4-1.3) | 0.28 |
| ≥ 92.9 ppb | 439 | 7 | 0.7 (0.3-1.6) | 0.44 |
| **Second trimester** |  |  |  |  |
| No exposure | 5191 | 108 | 1.0 (ref.) |  |
| >0-<44.8 ppb | 2781 | 77 | 1.3 (1.0-1.8) | 0.06 |
| ≥44.8-<78.7 ppb | 1395 | 27 | 0.9 (0.6-1.4) | 0.74 |
| ≥78.7-<94.8 ppb | 835 | 22 | 1.3 (0.8-2.0) | 0.32 |
| ≥ 94.8 ppb | 542 | 12 | 1.1 (0.6-1.9) | 0.84 |
| **Third trimester** |  |  |  |  |
| No exposure | 4186 | 99 | 1.0 (ref.) |  |
| >0-<44.0 ppb | 3254 | 76 | 1.0 (0.7-1.3) | 0.94 |
| ≥44.0 -<69.2 ppb | 1659 | 36 | 0.9 (0.6-1.3) | 0.66 |
| ≥69.2-<93.2 ppb | 978 | 21 | 0.9 (0.6-1.5) | 0.69 |
| ≥ 93.2 ppb | 667 | 14 | 0.9 (0.5-1.6) | 0.68 |

**Additional file 3B. Term low birth weight and average TCE exposure, by trimesters, Camp Lejeune, 1968-1985**

| **Exposure** | **Term low birth weight** | | **OR (95% CI)** | **p value** |
| --- | --- | --- | --- | --- |
|  | **No**  **#** | **Yes #** |  |  |
| **First trimester** |  |  |  |  |
| No exposure | 5586 | 114 | 1.0 (ref.) |  |
| >0-<2.0 ppb | 2619 | 72 | 1.3 (1.0-1.8) | 0.05 |
| ≥2.0-<3.9 ppb | 1298 | 28 | 1.1 (0.7-1.6) | 0.79 |
| ≥3.9-<16.1 ppb | 725 | 17 | 1.1 (0.7-1.9) | 0.60 |
| ≥ 16.1 ppb | 516 | 15 | 1.4 (0.8-2.5) | 0.20 |
| **Second trimester** |  |  |  |  |
| No exposure | 4134 | 78 | 1.0 (ref.) |  |
| >0-<2.3 ppb | 3296 | 80 | 1.3 (0.9-1.7) | 0.12 |
| ≥2.3-<3.9 ppb | 1653 | 40 | 1.3 (0.9-1.9) | 0.21 |
| ≥3.9-<20.3 ppb | 997 | 28 | 1.5 (1.0-2.3) | 0.07 |
| ≥ 20.3 ppb | 664 | 20 | 1.6 (1.0-2.6) | 0.07 |
| **Third trimester** |  |  |  |  |
| No exposure | 2910 | 67 | 1.0 (ref.) |  |
| >0-<2.0 ppb | 3893 | 87 | 1.0 (0.7-1.3) | 0.86 |
| ≥2.0-<3.9 ppb | 2062 | 47 | 1.0 (0.7-1.4) | 0.96 |
| ≥3.9-<16.8 ppb | 1099 | 31 | 1.2 (0.8-1.9) | 0.36 |
| ≥ 16.8 ppb | 780 | 14 | 0.8 (0.4-1.4) | 0.40 |

**Additional file 3C. Term low birth weight and average benzene exposure, by trimesters, Camp Lejeune, 1968-1985**

| **Exposure** | **Term low birth weight** | | **OR (95% CI)** | **p value** |
| --- | --- | --- | --- | --- |
|  | **No**  **#** | **Yes #** |  |  |
| **First trimester** |  |  |  |  |
| No exposure | 9289 | 203 | 1.0 (ref.) |  |
| >0-<1 ppb | 948 | 28 | 1.4 (0.9-2.0) | 0.13 |
| ≥ 1 ppb | 507 | 15 | 1.4 (0.8-2.3) | 0.26 |
| **Second trimester** |  |  |  |  |
| No exposure | 8949 | 192 | 1.0 (ref.) |  |
| >0-<1 ppb | 1021 | 30 | 1.4 (0.9-2.0) | 0.11 |
| ≥ 1 ppb | 774 | 24 | 1.4 (0.9-2.2) | 0.09 |
| **Third trimester** |  |  |  |  |
| No exposure | 8590 | 198 | 1.0 (ref.) |  |
| >0-<1 ppb | 1283 | 23 | 0.8 (0.5-1.2) | 0.26 |
| ≥ 1 ppb | 871 | 25 | 1.2 (0.8-1.9) | 0.31 |

**Additional file 4A. Birth weight and average PCE exposure*, by trimesters, term births, Camp Lejeune, 1968-1985**

| Exposure | # | Mean birth weight difference in grams (95% CI) | p value |
| --- | --- | --- | --- |
| **First Trimester** |  |  |  |
| Unexposed (ref) | 6529 | -- |  |
| >0 - <44.4 ppb | 2222 | -23.2 (-45.7, -0.7) | 0.04 |
| ≥44.4- <69.3 ppb | 1123 | -9.9 (-39.6, 19.8) | 0.51 |
| ≥69.3 - <92.9 ppb | 670 | 19.4 (-18.1, 57.0) | 0.31 |
| ≥92.9 ppb | 446 | -10.4 (-55.5, 34.7) | 0.65 |
| **Second Trimester** |  |  |  |
| Unexposed (ref) | 5299 | -- |  |
| >0 - <44.8 ppb | 2858 | -25.9 (-47.3, -4.6) | 0.02 |
| ≥44.8- <78.7 ppb | 1422 | -29.8 (-57.3, -2.4) | 0.03 |
| ≥78.7 - <94.8 ppb | 857 | -15.0 (-49.2, 19.2) | 0.39 |
| ≥94.8 ppb | 554 | -1.3 (-42.5, 40.0) | 0.95 |
| **Third Trimester** |  |  |  |
| Unexposed (ref) | 4285 | -- |  |
| >0 - <44.0 ppb | 3330 | -13.9 (-35.2, 7.4) | 0.20 |
| ≥44.0- <69.2 ppb | 1695 | -6.8 (-33.3, 19.7) | 0.62 |
| ≥69.2 - <93.2 ppb | 999 | 12.4 (-20.3, 45.2) | 0.46 |
| ≥93.2 ppb | 681 | -4.7 (-43.1, 33.6) | 0.81 |

* adjusted for prenatal care, sex of child, mother’s race, mother’s age, mother’s education, parity, previous fetal death, father’s age, and rank

**Additional file 4B. Birth weight and average TCE exposure*, by trimesters, term births, Camp Lejeune, 1968-1985**

| Exposure | # | Mean birth weight difference in grams (95% CI) | p value |
| --- | --- | --- | --- |
| **First Trimester** |  |  |  |
| Unexposed (ref) | 5700 | -- |  |
| >0 - <2.0 ppb | 2691 | -25.3 (-46.7, -3.9) | 0.02 |
| ≥2.0- <3.9 ppb | 1326 | 7.2 (-20.8, 35.3) | 0.61 |
| ≥3.9 - <16.1 ppb | 742 | -17.1 (-52.7, 18.5) | 0.35 |
| ≥16.1 ppb | 531 | -51.2 (-92.5, -9.9) | 0.02 |
| **Second Trimester** |  |  |  |
| Unexposed (ref) | 4212 | -- |  |
| >0 - <2.3 ppb | 3376 | -47.1 (-68.1, -26.0) | <0.01 |
| ≥2.3- <3.9 ppb | 1693 | -35.9 (-62.2, -9.5) | 0.01 |
| ≥3.9 - <20.3 ppb | 1025 | -57.0 (-88.7, -25.3) | <0.01 |
| ≥20.3 ppb | 684 | -50.1 (-87.5, -12.5) | 0.01 |
| **Third Trimester** |  |  |  |
| Unexposed (ref) | 2977 | -- |  |
| >0 - <2.0 ppb | 3980 | -39.5 (-61.6, -17.5) | <0.01 |
| ≥2.0- <3.9 ppb | 2109 | -15.8 (-41.8, 10.2) | 0.23 |
| ≥3.9 - <16.8 ppb | 1130 | -45.3 (-77.1, -13.5) | 0.01 |
| ≥16.8 ppb | 794 | -92.9 (-129.4, -56.5) | <0.01 |

*adjusted for sex of child, mother’s race, and parity

**Additional file 4C. Birth weight and average benzene exposure*, by trimesters, term births, Camp Lejeune, 1968-1985**

| Exposure | # | Mean birth weight difference in grams (95% CI) | p value |
| --- | --- | --- | --- |
| **First Trimester** |  |  |  |
| Unexposed (ref) | 9492 | -- |  |
| >0 - <1 ppb | 976 | -0.8 (-31.3, 29.7) | 0.96 |
| ≥1 ppb | 522 | -44.2 (-85.2, -3.2) | 0.03 |
| **Second Trimester** |  |  |  |
| Unexposed (ref) | 9141 | -- |  |
| >0 - <1 ppb | 1051 | -26.4 (-55.9, 3.2) | 0.08 |
| ≥1 ppb | 798 | -47.4 (-81.2, -13.7) | 0.01 |
| **Third Trimester** |  |  |  |
| Unexposed (ref) | 8788 | -- |  |
| >0 - <1 ppb | 1306 | -16.6 (-43.6, 10.4) | 0.23 |
| ≥1 ppb | 896 | -40.2 (-72.3, -8.1) | 0.01 |

* adjusted for prenatal care, sex of child, mother’s race, rank, and parity
