# Supplementary material for: Evaluation of contaminated drinking water and preterm birth, small for gestational age, and birth weight at Marine Corps Base Camp Lejeune, North Carolina: a cross-sectional study
Source: Environ Health. 2014 Nov 20;13:99. doi: 10.1186/1476-069X-13-99 (PMC4247681; doi:10.1186/1476-069X-13-99)
Supplement: Supplementary file 2 — Additional file 2: Figures S1-S5: Splines of selected outcomes and exposures. (DOCX 108 KB) [file 12940_2014_799_MOESM2_ESM.docx]

**Additional files – Figures 1-5**

**Evaluation of contaminated drinking water and preterm birth, small for gestational age, and birth weight at Marine Corps Base Camp Lejeune, North Carolina: A cross-sectional study**

Perri Zeitz Ruckart, Frank J. Bove, Morris Maslia

Figure 1. Average Monthly TCE Exposure, Entire Pregnancy, and

Small for Gestational Age

Odds Ratio


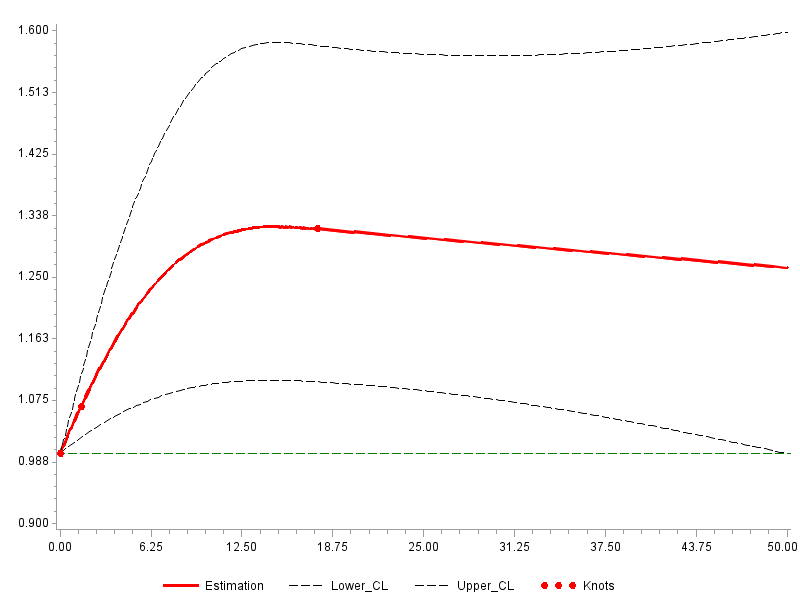


Average Monthly Exposure to TCE, Entire Pregnancy (µg/L)

Distribution of TCE (µg/L):

25th percentile = 0.20

Median = 1.44

Mean (SD) = 3.34 (13.06)

75th percentile = 2.72

95th percentile = 17.73

97.5th percentile = 21.53

Maximum = 508.23

Figure 2. Average Monthly 2^st^ Trimester Exposure to PCE and Preterm

Birth

Odds Ratio


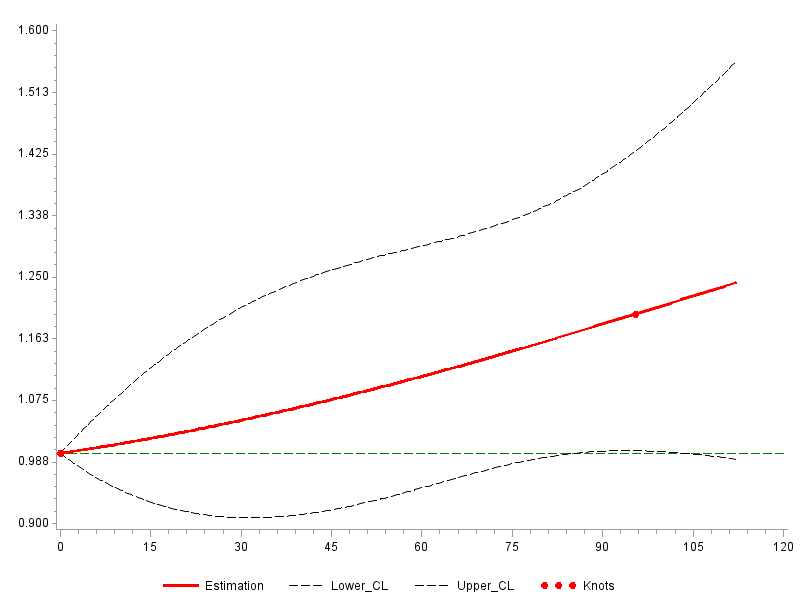


Average Monthly 2^nd^ Trimester Exposure to PCE (µg/L)

Distribution of PCE (µg/L):

Distribution of avg_PCEtrim2:

25th percentile = 0

Median = 0.0323333333

Mean (SD) = 26.425357879 (35.484225019)

75th percentile = 45.369333333

95th percentile = 95.427

97.5th percentile = 100.14066667

Maximum = 144.36

Figure 3. Average Monthly 2^nd^ Trimester Exposure to TCE and

Low Birth Weight Among Term Births

Odds Ratio

**
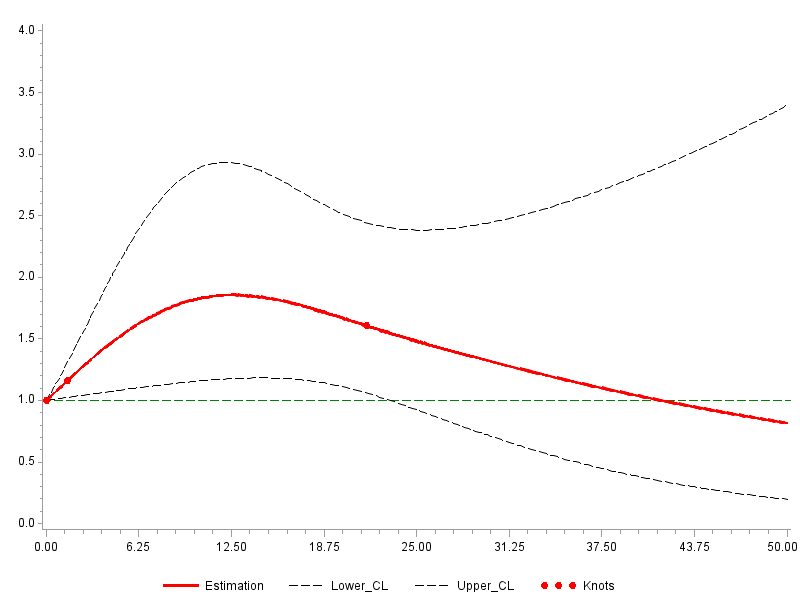
**

Average Monthly 2^nd^ Trimester Exposure to TCE (µg/L)

Distribution of TCE (µg/L):

25th percentile = 0

Median = 1.45

Mean (SD) = 3.61 (14.81)

75th percentile = 2.99

95th percentile = 21.60

97.5th percentile = 25.01

Maximum = 562.68

Figure 4. Average Monthly Exposure to Benzene, Entire Pregnancy, and

Low Birth Weight Among Term Births

Odds Ratio


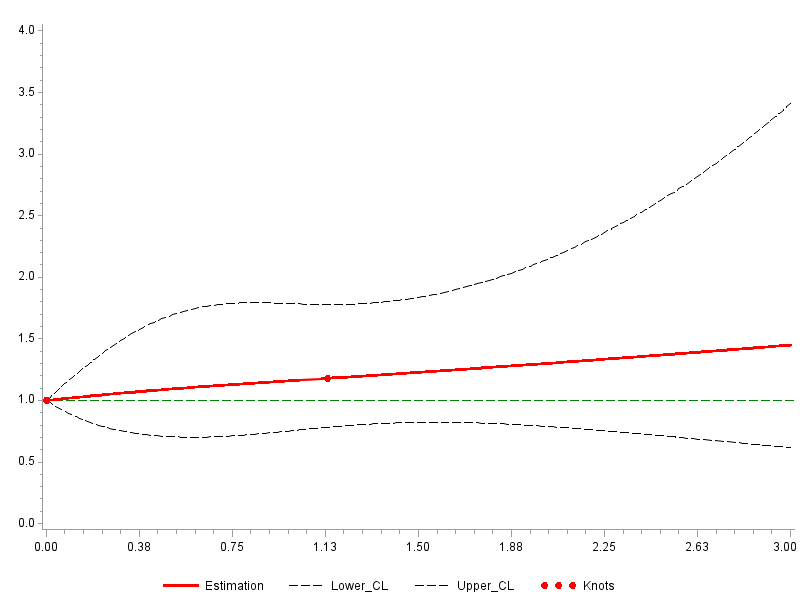


Average Monthly Exposure to Benzene, Entire Pregnancy (µg/L)

Distribution of Benzene (µg/L):

25th percentile = 0

Median = 0

Mean (SD) = 0.14 (0.45)

75th percentile = 0.01

95th percentile = 1.13

97.5th percentile = 1.648

Maximum = 8.57

Figure 5. Average Monthly Exposure to TCE, Entire Pregnancy, and Birth

Weight Among Term Births

Mean difference in grams


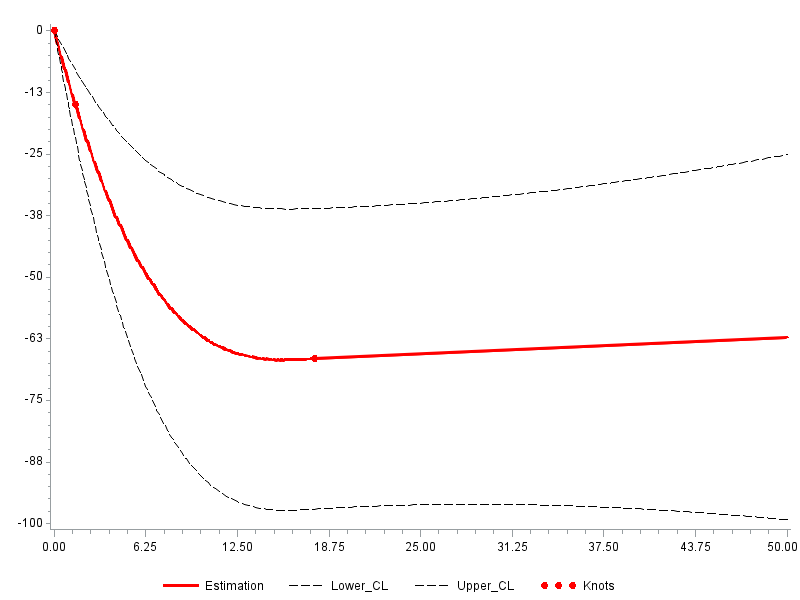


Average Monthly Exposure to TCE, Entire Pregnancy (µg/L)

Distribution of TCE (µg/L):

25th percentile = 0.20

Median = 1.43

Mean (SD) = 3.38 (13.50)

75th percentile = 2.71

95th percentile = 17.78

97.5th percentile = 21.55

Maximum = 508.23
